# New SmAP_F_ Mesogens Designed for Analog Electrooptics Applications

**DOI:** 10.3390/ma10111284

**Published:** 2017-11-09

**Authors:** Eva D. Korblova, Edward Guzman, Joseph E. Maclennan, Matthew A. Glaser, Renfan Shao, Edgardo Garcia, Yongqiang Shen, Rayshan Visvanathan, Noel A. Clark, David M. Walba

**Affiliations:** 1 Department of Chemistry and Biochemistry, University of Colorado, 215 UCB, Boulder, CO 80309-0215, USA; edward.guzman@colorado.edu (Edw.G.); edgardo.garcia@colorado.edu (Edg.G.); 2 Soft Materials Research Center, Department of Physics, University of Colorado Boulder, 390 UCB, Boulder, CO 80309-0390, USA; jem@colorado.edu (J.E.M.); matthew.glaser@colorado.edu (M.A.G.); shaor@colorado.edu (R.S.); sheny@colorado.edu (Y.S.); rayshan.visvanathan@colorado.edu (R.V.); Noel.Clark@Colorado.EDU (N.A.C.); 3 Department of Physics, University of Colorado, 390 UCB, Boulder, CO 80309-0390, USA; 4 Departamento De Quimica, Universidade De Brasilia, Brasilia DF 70910-900, Brazil

**Keywords:** ferroelectric liquid crystals, bent-core liquid crystals, polar and achiral liquid crystal phase, SmAP_F_ phase

## Abstract

We have previously reported the first realization of an orthogonal ferroelectric bent-core SmAP_F_ phase by directed design in mesogens with a single tricarbosilane-terminated alkoxy tail. Given the potentially useful electrooptic properties of this phase, including analog phase-only electrooptic index modulation with optical latching, we have been exploring its “structure space”, searching for novel SmAP_F_ mesogens. Here, we report two classes of these—the first designed to optimize the dynamic range of the index modulation in parallel-aligned cells by lowering the bend angle of the rigid core, and the second expanding the structure space of the phase by replacing the tricarbosilane-terminated alkyl tail with a polyfluorinated polyethylene glycol oligomer.

## 1. Introduction

Potential applications of the lamellar (smectic), tilted, polar liquid crystal phase (SmCP), the lamellar, orthogonal, polar phase SmAP, and the lamellar, orthogonal nonpolar phase (SmA) exhibited by bent-core liquid crystals (LCs) motivate continuing interest in their materials design and synthesis. For example, Jákli et al. have described a bistable “scattering” display mode of the SmC_S_P_A_ phase requiring no polarizers [1,2], O’Callaghan et al. reported fast analog electrooptic phase modulators with a SmC_A_P_F_ mesogen [3], and Takezoe et al. have reported an “ideal display mode” [4,5] obtained with certain bent-core SmA mesogens in what has become known as the SmAP_R_ phase.

**Figure 1:**
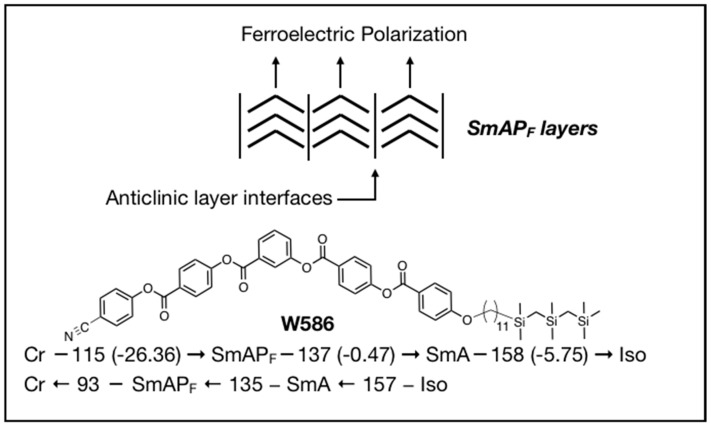
The chemical structure, phase sequence, transition temperatures (°C), and transition enthalpies (heating, kJ/mol) of **W586**, with an illustration of the SmAP_F_ layer structure showing the anticlinic layer interfaces leading to ferroelectric order in the bent-core SmAP_F_ phase.

The SmAP_F_ phase, an achiral, orthogonal phase with spontaneous ferroelectric polarization parallel to the smectic layers, was described in a theoretical exercise in the early 1990s [6], and first realized experimentally in an interesting (but very slow-switching) polymeric LC system composed of calamitic monomer units in 2004 [7]. A low molar mass SmAP_F_ bent-core mesogen, codenamed **W586** (Figure 1), obtained by directed design, was reported in 2011 [8,9], and a method for high-quality alignment of the SmAP_F_ phase has recently been described [10].

The approach for the design of structures exhibiting the SmAP_F_ phase involved structural modifications of a previously known antiferroelectric SmAP_A_ mesogen with a structure similar to **W586**, but lacking the tricarbosilane [11,12]. Our basic concept for obtaining the ferroelectric SmAP_F_ phase involved removing the generally strong preference for synclinic layer interfaces in smectics, which leads to antiferroelectric ordering of bent-core mesogens, an outcome thought to be due to out-of-layer fluctuations typically seen with mesogens possessing alkoxy tails [13]. The desired anticlinic layer interfaces were obtained by introducing functionality expected to suppress out-of-layer fluctuations—in the case of **W586**, the tricarbosilane terminating group (earlier applications of carbosilanes in smectic LCs can be found in [14,15]). This approach to suppression of the out-of-layer fluctuations removes an hypothesized entropic preference for synclinicity, but seemingly does not in itself favor anticlinic interfaces [16]. However, with the **W586** structure, incorporation of the tricarbosilane-terminated tail in fact produced the desired anticlinic inter-layer structure and ferroelectric order.

**Figure 2:**
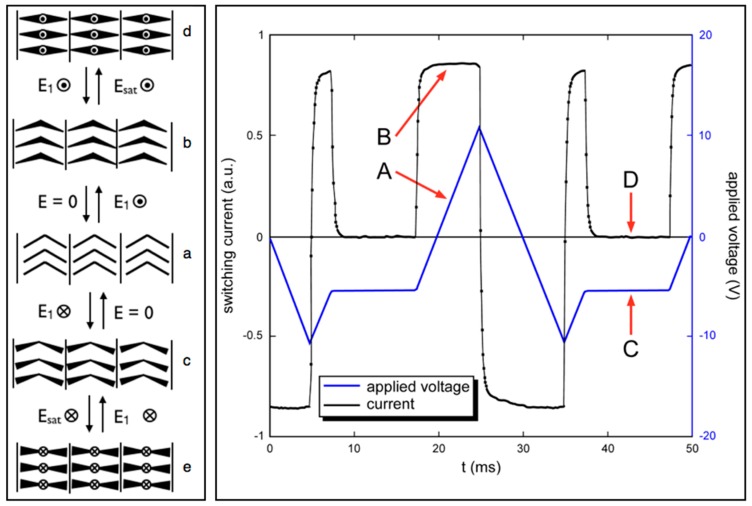
V-shaped electrooptic switching in the SmAP_F_ phase. (**Left**): Cartoon showing the bent-core polar axis reorientation occurring in a bookshelf-aligned cell (substrates parallel to the page) in response to applied electric fields: (**a**) Bookshelf layer structure with **P** parallel to the substrates at E = 0; (**b**,**c**) Partial reorientation of **P** with applied field E_1_ (sub-saturation); (**c**,**d**) At E_sat_ (saturation) with **P** along E; (**Right**): Electrostatic V-shaped switching with optical latching–A Applied voltage (blue) changing with time, B current response (black) to voltage ramp (constant current); D No current, and essentially no energy dissipation (latching), at constant applied voltage (C).

As previously reported [17], the electrooptic behaviour of **W586** is interesting. With rubbed polyimide-coated glass plates, the layers orient perpendicular to the substrates, with the ferroelectric polarization **P** parallel to the plates. Though there is effectively random planar alignment (a smectic focal conic texture is seen by polarized optical microscopy), application of fields normal to the plates induces no brush rotation, but causes a smooth, analog change in birefringence with increasing field until saturation, where the polarization is oriented normal to the plates, directly along the field (Figure 2, Left). This is an example of electrostatic V-shaped switching, as first reported for calamitic de Vries SmC* materials with high polarization, [18,19]. For the SmAP_F_ phase in uniformly aligned planar cells (see below), V-shaped switching gives uniform, phase-only modulation of incident polarized light, with no rotation of the optic axis in the cell, a potentially useful effect. This electrooptic mode gains added attractiveness in applications, since no power is required to maintain an optical state (i.e., the system exhibits optical latching) (Figure 2, Right) [20].

Potential applications of SmAP_F_ have led to further investigation. For example, as mentioned above, a method for obtaining high quality bookshelf alignment of **W586** in the SmAP_F_ phase with excellent azimuthal orientational control has recently been reported [10]. Here, we describe work aimed at exploring the structure space of the SmAP_F_, both to improve fundamental understanding of the chemical structural features leading to the phase, and with an eye to improving the electrooptic properties exhibited in this novel manifestation of electrostatic V-shaped switching.

## 2. Design Rationale for the New SmAP_F_ Mesogens

**Figure 3:**
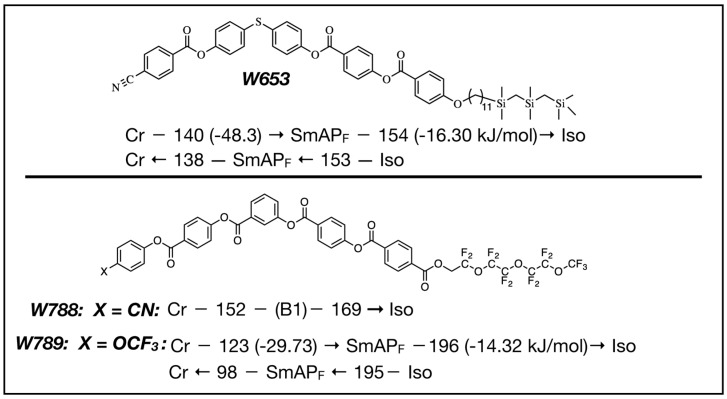
Structures, phase sequences, transition temperatures (°C) and transition enthalpies on heating (kJ/mol) by differential scanning calorimetry (DSC), of the new mesogens. The thio-bisphenol ester **W653** possesses an enantiotropic SmAP_F_ phase, which transitions directly to isotropic on heating. **W788**, the PF- PEG analog of **W586**, exhibits only a monotropic B1 (columnar) phase on cooling. **W789**, possessing a trifluoromethoxy tail in place of the cyano group of **W586**, exhibits a wide temperature range (almost 75 °C) enantiotropic SmAP_F_ phase. The details of the phase assignments are given below.

Prototypes of two new classes of SmAP_F_ mesogens are reported. In one of these, a 4,4′-thiobisphenol diester unit is incorporated into the mesogen core, while maintaining other key features of the **W586** structure, giving **W653** (Figure 3, top). The thioether core present in **W653** was chosen based upon the expectation that the bend angle for this bent-core material should be smaller than that of **W586** (~120°). Thus, in 4-4′-thiobisphenol (bis(4-hydroxyphenyl)sulfide) in the crystalline solid state, the OPh-S-PhO angle is reported to be 104.21° by single crystal X-ray crystallography [21], and the calculated angle in the gas phase (Spartan 14 at the MP2 6-31G* level) is 102°. The smaller bend angle at the center of the core was expected to decrease the “overall average bend angle” of the core, leading to an increase in the modulation depth of the index change on switching relative to **W586**, other things being equal.

The second new structural class, exemplified by **W788** and **W789**, (Figure 3, bottom) was chosen to explore the efficacy of polyfluorinated polyethyleneglycol (PF-PEG) tails as an alternative to the tricarbosilane-terminated alkoxy tail for suppression of out-of-layer fluctuations, thereby removing the normal preference for synclinicity, and allowing the desired anticlinic layer structure in the SmAP phase. This approach is motivated by a considerable body of research on calamitic ferroelectric liquid crystals (FLCs) driven by researchers at 3M Company in the 1990s, showing that the PF-PEG tails incorporated into calamitic FLC structures produce de Vries smectics [22], a phase also thought to be favored by suppression of out-of-layer fluctuations [13,23].

## 3. Synthesis of the New Mesogens

**Scheme 1:**
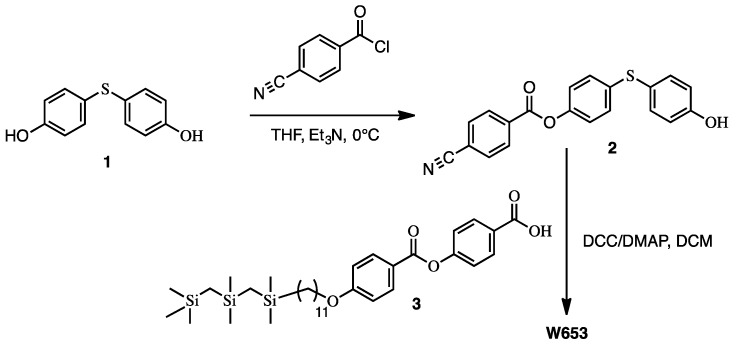
Synthesis of the diphenylthioether bent-core **W653**.

Diphenylthioether **W653** was readily prepared as indicated in Scheme 1. Synthesis of the diphenylthioether bent-core **W653**. Monoesterification of 4,4′-thiobisphenol (**1**) with 4-cyanobenzoyl chloride gave intermediate thioether **2**. Esterification of the second phenolic hydroxyl group with carboxylic acid **3** [24] using DCC/DMAP provided the target thioether **W653** in good yield.

**Scheme 2:**
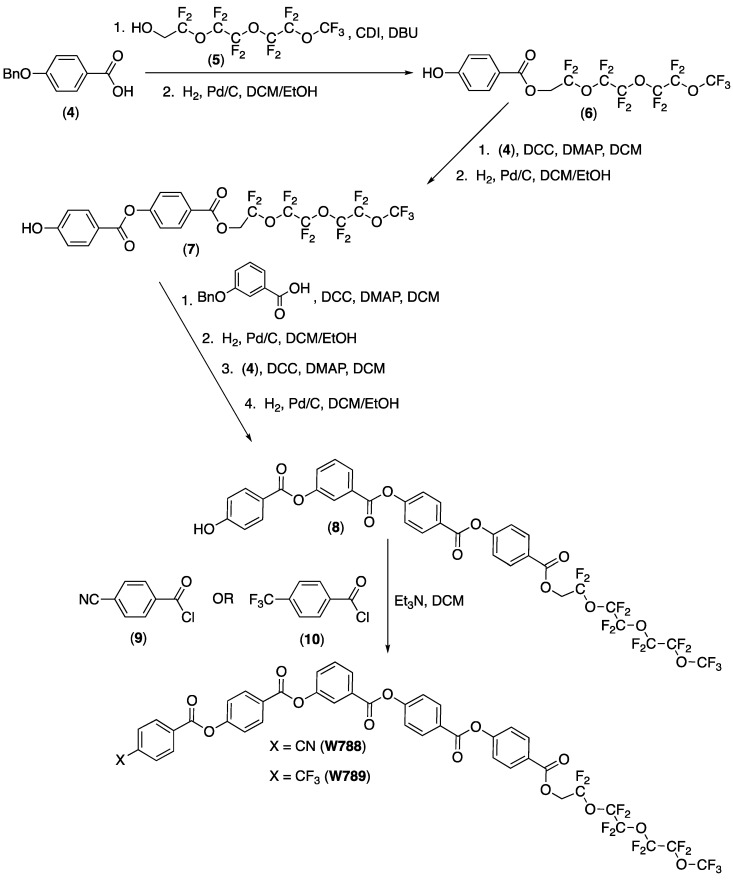
Synthesis of **W788** and **W789**.

Incorporation of a PF-PEG tail with commercially available fluorinated starting materials was accomplished using an ester linkage in place of a simple aryl ether, leading to structures **W788** and **W789**, possessing a cyano group, and trifluoromethoxy group, respectively, opposite the PF-PEG tails, as indicated in Scheme 2.

Both fluoro-PEG mesogens **W788** and **W789** were prepared from the key tetracyclic phenol intermediate (**8**). Synthesis of tetracyclic **8** relies upon prior work of Finkelmann, et al., who reported the synthesis of the PF-PEG phenol **6**. Thus, esterification of benzyl ether protected p-hydroxybenzoic acid (**4**) with the now commercially available hydroxymethylene-terminated PF-PEG **5**, using carbonyldiimidazole (CDI) and 1,8-Diazabicycloundec-7-ene (DBU), followed by debenzylation, gave Finkelmann’s phenol **6**. A second esterification with benzoic acid **4**, then debenzylation, gave phenol (**7**). Esterification of **7** with m-benzyloxybenzoic acid, debenzylation, then another esterification with p-benzyloxybenzoic acid (**4**) followed by debenzylation gave the key intermediate tetracyclic phenol **8**. Esterification of phenol **8** using either p-cyanobenzoyl chloride **9**, or p-trifluoromethoxybenzoyl chloride **10**, gave mesogens **W788** and **W789**, respectively.

## 4. Properties of Diphenylthioether W653

The phase sequences (obtained by polarized light microscopy) and transition temperatures and enthalpies (obtained by DSC) for the new mesogens are given in Figure 3. Gratifyingly, the diphenylthioether **W653** possessed the desired enantiotropic SmAP_F_ phase between 140 °C and 154 °C. Small-angle X-ray scattering shows a layer spacing of 57.0 Å at 140 °C, expanding to about 57.3 Å at 154 °C.

For SmAP mesogens such as these, possessing only one tail, the layer spacing was more difficult to interpret at a molecular level than for nominal calamitic smectics. A simple molecular mechanics fully extended conformation for **W653** was about 49 Å in length, suggesting a “partial bilayer” layer structure. This is also seen for the prototype SmAP_F_ material **W586**. Lacking results of high-end computational dynamic simulations (currently in progress for **W586**), it was not possible to understand the layer structure of **W653** in more detail.

**Figure 4:**
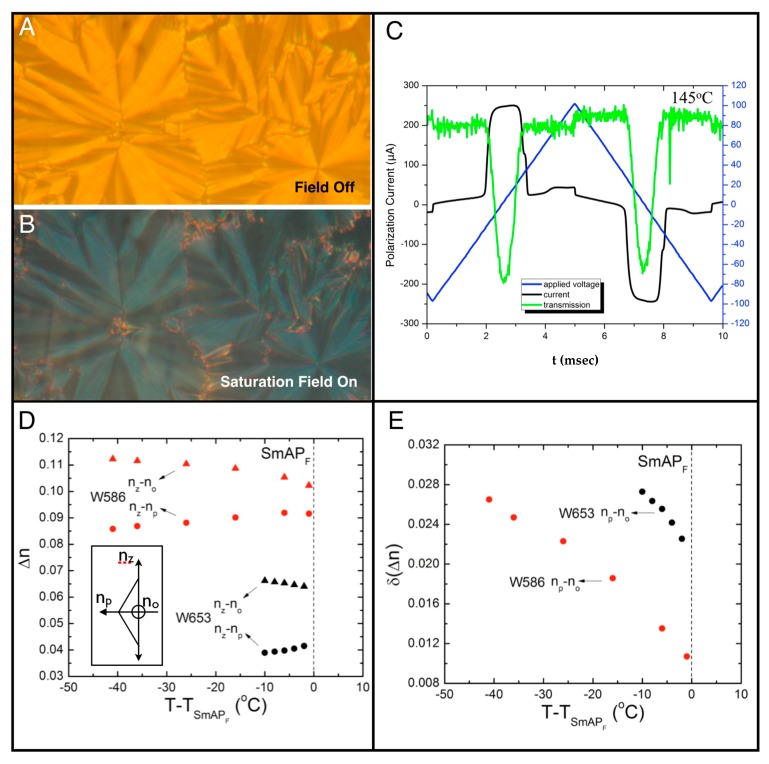
(**A**,**B**): Polarized light microscopic images (polarizer and analyzer crossed) of a random planar aligned indium-tin oxide (ITO)-glass cell (T = 140 °C) containing **W653** of sample thickness 8 µm, and rubbed polyimide (PI) insulating layers. As for **W586**, the rubbed PI gives random planar alignment of the smectic, but provides the dielectric behavior necessary for V-shaped switching. The SmAP_F_ phase of **W653** shows typical focal conic textures without field, and with an applied field at saturation. The field-on state shows the expected increase in ∆n; (**C**): Electrooptics of the SmAP_F_ phase, showing a flat current response and “V-shaped” transmission response to a voltage ramp. The green curves indicate transmission, with a minimum value close to zero. These are the defining characteristics of electrostatic V-shaped switching; (**D**,**E**): ∆n and δ∆n, as a of function of applied field (E = 0 and E > saturation) and temperature for **W586**, and **W653**.

As shown in Figure 4A,B, in planar-aligned electrooptic cells (rubbed polyimide on ITO-coated glass plates), the observed focal conic texture of **W653** exhibits the SmAP_F_ signature change in birefringence, without brush rotation, upon application of a field. In addition, electrostatic V-shaped switching behavior is clearly evidenced by the electrooptics data given in Figure 4C. The ferroelectric polarization derived from the observed polarization reversal current for **W653** ranges from 660 nC/cm^2^ at 154 °C to 715 nC/cm^2^ at 140 °C. This large observed polarization is similar to the maximum ferroelectric polarization observed for the SmAP_F_ prototoype **W586** of 587 nC/cm^2^ [8].

Defining the three refractive indices n_Z_, n_P_, and n_O_, in a planar-aligned SmAP_F_ cell as indicated in the inset, the index seen for the field off state (**P** parallel to the plates) is (n_Z_-n_P_), while that seen for the field on state (at saturation) is (n_Z_-n_O_). The index modulation for the field off/on states is greater for **W653** than for **W586**, as indicated in Figure 4D, consistent with the expectation that a tighter bend angle would increase the maximum modulation depth in the V-shaped switching by increasing n_P_. The higher δ(∆n) of **W653** relative to the prototype **W586** at the same reduced temperatures, is illustrated in Figure 4E.

## 5. Properties of a New SmAP_F_ Mesogen Possessing a PF-PEG Tail (W789)

As discussed above, the first known SmAP_F_ mesogens were designed to test the hypothesis that suppression of out-of-layer fluctuations allows the formation of anticlinic layer interfaces (ferroelectric order for bent-cores) in smectic LCs. In the case of the classic SmAP_F_ mesogen **W586**, and the more strongly bent diphenylthioether **W653**, suppression of out-of-layer fluctuations was achieved by incorporating a tricarbosilane moiety at the end of the tail, expected to enhance nano-phase segregation at the layer interfaces, thereby allowing the formation of anti-clinic layer interfaces.

**Figure 5:**
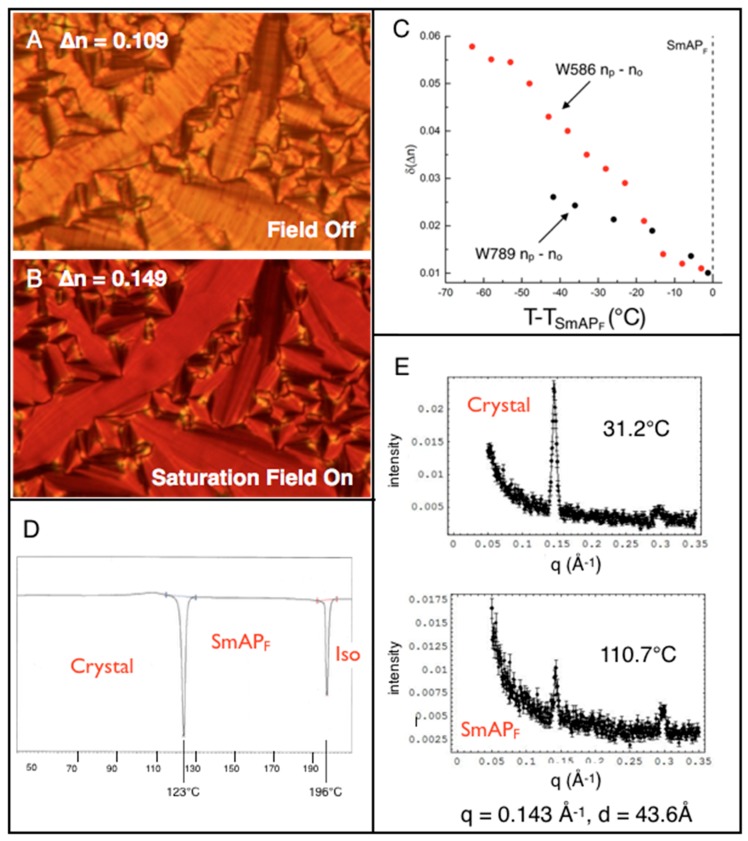
(**A**,**B**) Polarized light micrographs (polarizer and analyzer crossed) of a random planar aligned ITO-glass cell (T = 150 °C) containing **W789**. The cell was 4.05 µm thick, with rubbed polyimide (PI) insulating layers; (**C**) δ(∆n), as a function of applied electric field (zero or >saturation) and reduced temperature for **W586** and **W789**. In this case, **W586** possesses a larger modulation depth δ∆n than the new mesogen **W789**; (**D**) DSC scan on a sample of **W789** on heating at 5.0 °C/min; (**E**) Small-angle X-ray scattering from the crystal phase (top), and from the SmAP_F_ phase (on cooling–monotropic at this temperature) (bottom).

Here we provide an additional positive test of this hypothesis with characterization of a SmAP_F_ mesogen possessing a PF-PEG tail. The first material synthesized for this effort, **W788** (Figure 3), possesses a cyano group “opposite” the PF-PEG tail, similar to **W586** and **W653**. Somewhat surprisingly, **W788** did not provide a SmAP phase, but rather a monotropic B1 phase, as indicated in Figure 3. The B1 phase is a variety of columnar phase well known among bent-core FLCs. However, when the cyano group opposite the PF-PEG tail was replaced by another well-known “short” polar group, trifluoromethoxy, a new SmAP_F_ mesogen, **W789**, resulted. The phase sequence, transition temperatures, and transition enthalpies on heating obtained from DSC are given in Figure 3. As indicated, **W789** exhibits an enantiotropic SmAP_F_ phase between 123 °C and 196 °C. The phase assignment is based upon observation of the diagnostic electrooptics expected for the SmAP_F_ phase, analog index modulation with no brush rotation, as indicated in the photomicrographs given in Figure 5A,B. In this case the index modulation depth is slightly less than half that seen with **W586**, as illustrated in Figure 5C. For **W789** the maximum observed index modulation depth δ(∆n) (measured about 40 °C below the Iso–SmAP_F_ transition, just before crystallization) is about 0.025, while that for **W586** is 0.0425 at the same reduced temperature. It is interesting to note that we observe no change in the polarized light microscope texture upon crossing the Crystal–SmAP_F_ phase boundary. This led initially to characterization of the material at low temperature as a glass. However, the DSC (Figure 5D) shows a sharp first-order transition from the SmAP_F_ to the crystal, both on heating and on cooling, with a large transition enthalpy.

Additionally, the small-angle X-ray scattering profile of this crystal phase is essentially identical to that of the SmAP_F_, as shown in Figure 5E. Also notable is the prominent second harmonic of the layer peak, consistent with suppression of out-of-layer fluctuations. The observed layer peak at q = 0.143 Å^−1^ suggests a layer spacing in the SmAP_F_ phase of **W789** of d = 43.6 Å. The X-ray diffraction (XRD) results suggest that the structure of the crystal phase maintains the “smectic layer” metric, with apparent crystallization of the molecules in these layers.

Finally, as expected for a SmAP_F_ mesogen, **W789** exhibits a high ferroelectric polarization P = 786 nC/cm^2^ at 150 °C, and P = 837 nC/cm^2^ at the lower end of the SmAP_F_ phase temperature range. This value is to our knowledge the highest polarization seen to date in a SmAP_F_ mesogen, slightly higher than the previous record measured for **W623**, an isomer of **W586**, of 830 nC/cm^2^ [17].

## 6. Speculation Regarding the Layer Structure of the SmAP_F_ Phase of W789

**Figure 6:**
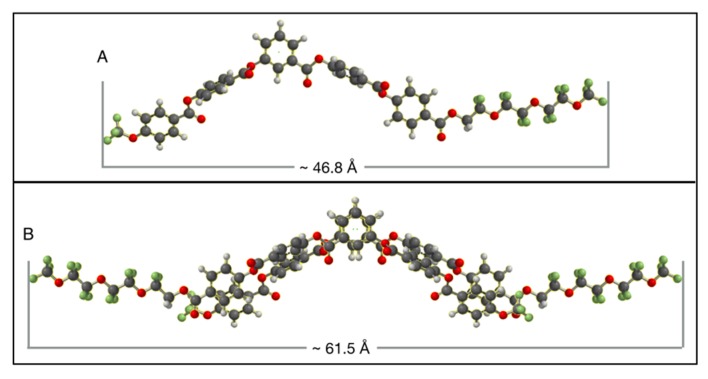
“Starting point” layer structure for **W789**. (**A**) “fully extended” conformation of a single molecule of **W789**; (**B**) Pair of **W586** molecules overlapping at the core.

As indicated in Figure 5, the layer spacing in the SmAP_F_ phase of **W789** obtained from X-ray diffraction is 43.6 Å. A “fully extended” conformation of this molecule imposing an “all anti” PF-PEG tail (not actually expected to be a well-populated conformation) as indicated in Figure 6A, is about 46.8 Å (including two-times Spartan’s fluorine atom Van der Waals radius)—seemingly a fairly close match. However, the picture is complicated by the fact that the mesogen has only one tail, and our “first wild guess” is that the cores should prefer to overlap giving the molecule pair indicated in Figure 6B. The “molecular length” for this pair is about 61.5 Å, which is much larger than the experimental layer spacing. This model forms the basis of the often-proposed “partial bilayer” structure, where the tails overlap considerably. While the body of work on PF-PEG conformational analysis is small, computations by Glaser et al. suggest that the gauche and anti conformations of perfluoroethyleneglycol are similar in energy [25], leading to a higher incidence of gauche conformers of the tail than would be expected for a nominal straight-chain hydrocarbon tail. This would tend to produce more “twisted” tails with a shorter length on average.

**Figure 7:**
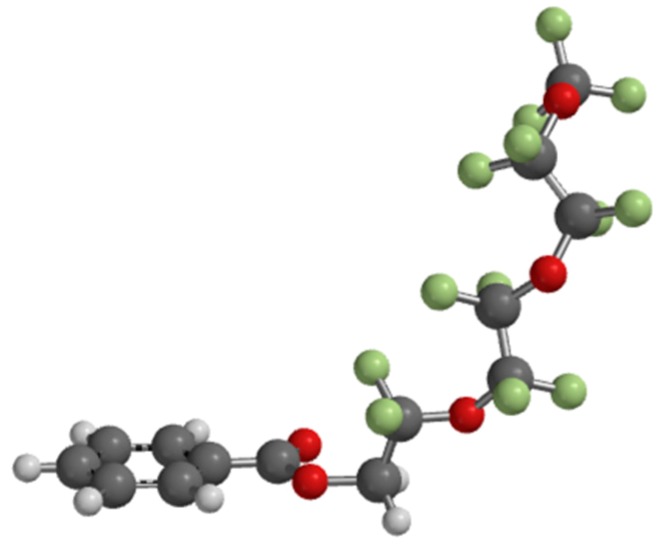
A low energy conformation of a benzoic acid ester of the PF-PEG tail of **W789**.

In addition, a preliminary computational conformational analysis of PhCOOCH_2_CF_2_OCF_2_CF_2_OCF_3_ using a “small basis set” density function model (ωB97X-D 6-31G*) in a “nonpolar solvent” (an attempt to negate strong intramolecular interactions), using the computational package Spartan’16 [26], gives an average value for the (carbonyl carbon-O-CH_2_-CF_2_-) dihedral angle for the 14 lowest energy conformers of 104°. Removing one outlier with energy in the middle of the range and a calculated dihedral that is close to 180°, gives an average dihedral of 98° for this molecular fragment. While these calculations should be considered a primitive starting point in the interesting conformational analysis of the Ph-PF-PEG system, if the indicated dihedral angle is indeed averaging about 100° in the LC phase, then “bent” conformations such as that shown in Figure 7 would be preferred. It is easy to imagine that a combination of “partial bilayer” tail overlap and a large majority of twisted and bent conformations could lead to the observed layer spacing.

## 7. Conclusions

Two new chemical structural classes exhibiting the interesting and relatively rare SmAP_F_ liquid crystal phase are reported. One of these, the thio-bisphenol derivative **W653**, shows enhanced electrooptic index modulation depth relative to the prototype SmAP_F_ mesogen **W586**, by a design accomplished by lowering the bend angle of the mesogen with a diphenylthioether core. In addition, the occurrence of this phase in a mesogen possessing a PF-PEG tail significantly expands the SmAP_F_ phase chemical structural space, and is consistent with the hypothesis that suppression of out-of-layer fluctuations can allow the formation of anticlinic layer interfaces, leading to a ferroelectric layer structure for a bent-core mesogen. The discovery of new SmAP_F_ structural classes suggests it may be possible to obtain such materials with lowered SmAP_F_ phase temperature ranges, allowing realistic exploration of potential applications.

## 8. Synthesis Experimental Workflow

Reagents and starting materials were used as purchased from qualified suppliers without additional purification. Tetrahydrofuran (THF) was freshly distilled under argon from sodium benzophenone ketyl, dichloromethane was purchased from Sigma-Aldrich^®^ (Now Millipore Sigma, Darmstadt, Germany) dry, sure seal grade solvent, and other solvents were used as purchased. Non-aqueous reactions were performed in oven-dried glassware under an atmosphere of dry argon. Purification by flash chromatography was performed with silica gel (40–63 microns) purchased from Zeochem AG^®^. (Uetikon am See, Switzerland) Analytical thin-layer chromatography (TLC) was performed on Silica gel 60 F_254_ TLC plates from Millipore Sigma (Darmstadt, Germany) Compounds were visualized with shortwave ultra-violet (UV), or by staining with I_2_. Nuclear magnetic resonance (NMR) spectra were obtained with a Bruker Avance-III 300 spectrometer (Brooker Daltonics Inc., Bellerica, MA, USA) or with a Varian INOVA 500 spectrometer (no longer commercially available). NMR chemical shifts were referenced to CHCl_3_ (7.24 ppm for ^1^H, 77.16 ppm for ^13^C). Exact mass was determined using electrospray ionization time-of-flight (ESI-TOF) mass spectrometry. Liquid crystal phase sequences and phase transition temperatures were determined using polarized light microscopy with a Nikon Optiphot 2 POL microscope equipped with an (Instec temperature-controlled hot stage. DSC was performed using a Mettler DSC823^e^ differential scanning calorimeter (Mettler Toledo, Columbus, OH, USA). Electrooptics and birefringence measurements made using a Zeiss microscope (Carl Zeiss Microscopy, LLC, Thornwood, NY, USA) fitted with an Ehringhaus rotary compensator with quartz combination plates and 656.3 nm light.

**4-[(4-hydroxyphenyl)sulfanyl]phenyl 4-cyanobenzoate (2)**

4-[(4-hydroxyphenyl)sulfanyl]phenol (**1**) (6.60 g, 30 mmol) was dissolved in THF (200 mL), and cooled to 0 °C. Triethylamine (3.64 g, 36 mmol, 5 mL) was then added, followed by dropwise addition of 4-cyanobenzoyl chloride (5.00 g, 30 mmol) in THF (20 mL). The reaction mixture was then allowed to stir overnight at room temperature, then poured to a saturated aqueous solution of NH_4_Cl (200 mL) and extracted with 3 aliquots of EtOAc. The combined organic layers were washed with water, brine, dried over MgSO_4_, filtered and concentrated at reduced pressure. The crude product was purified by flash chromatography (silica gel, CH_2_Cl_2_/1% EtOH), to afford ester **2** as a white solid (3.60 g. 35%).

^1^H NMR (300 MHz, Chloroform-d) ∂ ppm: 8.35–8.22 (m, 2H), 7.88–7.75 (m, 2H), 7.47–7.33 (m, 2H), 7.30–7.15 (m, 2H), 7.15–7.04 (m, 2H), 6.90–6.77 (m, 2H), 5.06 (s, 1H). ^13^C NMR (75 MHz, CDCl_3_) ∂ ppm: 163.77, 156.28, 148.73, 136.88, 135.88, 133.36, 132.56, 130.79, 129.43, 124.27, 122.12, 117.95, 117.21, 116.75.

**4-[(4-{4-[4-({11-[({dimethyl[(trimethylsilyl)methyl]silyl}methyl)dimethylsilyl]undecyl}oxy)-benzoyloxy]benzoyloxy}phenyl)sulfanyl]phenyl 4-cyanobenzoate (W653)**

To a solution of 4-[4-({11-[({dimethyl[(trimethylsilyl)methyl]silyl}methyl)dimethylsilyl]-undecyl}oxy)benzoyloxy]benzoic acid (3) [7] (0.905 g, 0.14 mmol) and ester (2) in CH_2_Cl_2_ (100 mL), DCC was added (0.50 g, 0.24 mmol) as well as a trace of DMAP. The reaction mixture was allowed to stir at room temperature for 2 days, then filtered, washed sequentially with water, 5% CH_3_COOH, water, and brine, dried over MgSO_4_, filtered, and concentrated at reduced pressure. The resulting crude product was purified by flash chromatography (silica gel, CH_2_Cl_2_). The solvent was removed at reduced pressure, filtered, recrystallized from CH_3_CN/EtOAc (40:10), and dried in vacuo overnight, giving 0.712 g, (53%) of **W653**.

**DSC 5 (deg/min ramp):**

Heating: X − 136.3 °C→SmAP_F_ (ΔH = −16.30 KJ mol^−1^) − 150.1 °C→I (ΔH = −15.57 KJ mol^−1^).

Cooling: I − 147.7 °C→SmAP_F_ (ΔH = 16.10 KJ mol^−1^) − 133.2 °C→X (ΔH = 15.46 KJ mol^−1^).

^1^H NMR (300 MHz, Chloroform-d) ∂ ppm: 8.36–8.21 (m, 4H), 8.21–8.10 (m, 2H), 7.88–7.77 (m, 2H), 7.52–7.32 (m, 6H), 7.27–7.13 (m, 4H), 7.05–6.93 (m, 2H), 4.05 (t, *J* = 6.5 Hz, 2H), 1.83 (p, *J* = 6.7 Hz, 2H), 1.55–1.28 (m, 16H), 0.49 (d, *J* = 8.5 Hz, 2H), 0.11–0.02 (m, 21H), 0.27 (d, *J* = 7.0 Hz, 4H). ^13^C NMR (75 MHz, CDCl_3_) ∂ ppm: 164.46, 164.44, 164.01, 163.55, 155.67, 150.52, 149.74, 134.21, 133.33, 132.89, 132.68, 132.58, 132.05, 131.99, 130.82, 126.73, 122.90, 122.48, 122.33, 121.03, 117.95, 117.30, 114.58, 68.56, 33.87, 29.79, 29.76, 29.72, 29.55, 29.53, 29.24, 26.14, 24.14, 18.22, 5.94, 4.18, 2.62, 1.63, −0.28.

HRMS (ESI-TOF) *m*/*z* for C_54_H_67_NO_7_Si_3_SLi^+^ [M + Li]^+^; calc. 964.4101, found 964.4089.

**2,2-difluoro-2-{1,1,2,2-tetrafluoro-2-[1,1,2,2-tetrafluoro-2-(trifluoromethoxy)ethoxy]ethoxy}ethyl 4-(benzyloxy)benzoate**

Starting material 2,2-difluoro-2-{1,1,2,2-tetrafluoro-2-[1,1,2,2-tetrafluoro-2-(trifluoromethoxy)-ethoxy]ethoxy}ethan-1-ol (**5**) was as purchased from Exfluor^®^ (Round Rock, TX, USA) Compound (**6**) was prepared by a modification of the procedure described in [27], using CDI in the presence of DBU as coupling reagent, yield 57% (lit. 86%).

**4-(4,4,6,6,7,7,9,9,10,10,12,12,12-tridecafluoro-2,5,8,11-tetraoxadodecanoyl)phenyl 4-(benzyloxy)benzoate**

To a solution of 2,2-difluoro-2-{1,1,2,2-tetrafluoro-2-[1,1,2,2-tetrafluoro-2-(trifluoromethoxy)-ethoxy]ethoxy}ethyl 4-hydroxybenzoate (**6**) (11.72 g, 23 mmol) and 4-benzyloxy benzoic acid (**4**) (5.16 g, 23 mmol) in CH_2_Cl_2_ (136 mL) DCC was added, (5.7 g, 28 mmol) as well as a trace of DMAP. The reaction mixture was allowed to stir at room temperature for 3 days, then filtered, washed with water, 5% CH_3_COOH, water, brine, dried over MgSO_4_, filtered, and concentrated at reduced pressure. The crude product was purified by flash chromatography (silica gel, CH_2_Cl_2_). White solid (11.88 g, 71%). Glass 128.7–130 °C SmA 161–163.5 °C I.

^1^H NMR (300 MHz, Chloroform-d) ∂ ppm: 8.22–8.07 (m, 4H), 7.51–7.27 (m, 7H), 7.14–7.02 (m, 2H), 5.17 (s, 2H), 4.72 (t, *J* = 9.3 Hz, 2H).

^13^C NMR (75 MHz, CDCl_3_) ∂ ppm: 164.28, 164.24, 163.48, 155.71, 136.16, 132.61, 131.73, 128.88, 128.47, 127.64, 125.81, 122.29, 121.55, 114.97, 70.38, 62.61, 62.16, 61.72.

**4-(4,4,6,6,7,7,9,9,10,10,12,12,12-tridecafluoro-2,5,8,11-tetraoxadodecanoyl)phenyl 4-hydroxy-benzoate (7)**

A solution of the benzyl ether-protected phenol from above (25.00 g, 34 mmol) in CH_2_Cl_2_ (400 mL) and EtOH (150 mL) was first briefly evacuated and purged with argon, then 10% Pd/C catalyst (4 g) was added. The argon atmosphere was replaced by hydrogen gas, and the reaction mixture was stirred at room temperature for 8 hours. Hydrogen was pumped out of the system and the flask was purged thoroughly with argon. The mixture was filtered through Celite and solvents were removed under reduced pressure. This gave product as a white crystalline powder, m.p. 117–119.5 °C (21.60 g, 99%).

^1^H NMR (300 MHz, Chloroform-d) ∂ ppm: 8.19–8.03 (m, 4H), 7.39–7.27 (m, 2H), 6.99–6.85 (m, 2H), 6.13 (s, 1H), 4.72 (t, *J* = 9.3 Hz, 2H). ^13^C NMR (75 MHz, CDCl_3_) ∂ ppm: 164.44, 164.31, 160.79, 155.65, 132.93, 131.78, 125.87, 122.31, 121.53, 115.71, 62.66, 62.21, 61.76.

**4-{[4-(4,4,6,6,7,7,9,9,10,10,12,12,12-tridecafluoro-2,5,8,11-tetraoxadodecanoyl)phenoxy]carbonyl}-phenyl 3-(benzyloxy)benzoate**

To a solution of 3-benzyloxy benzoic acid and phenol **7** resulting from the prior debynzylation (6.26 g, 25 mmol) (15.95 g, 25 mmol) was added was added DCC (5.70 g, 28 mmol) and trace of DMAP in CH_2_Cl_2_ (400 mL). The reaction mixture was allowed to stir at room temperature for 3 days, then filtered, washed with water, 5% CH_3_COOH, water, brine, dried over MgSO_4_, filtered, and concentrated at reduced pressure. The resulting product was purified by flash chromatography (silica gel, CH_2_Cl_2_), followed by crystallization from CH_3_CN. White solid, I 130 °C SmA 66 °C glass (17.54 g, 83%).

^1^H NMR (300 MHz, Chloroform-d) ∂ ppm: 8.37–8.23 (m, 2H), 8.23–8.08 (m, 2H), 7.93–7.78 (m, 2H), 7.55–7.24 (m, 11H), 5.16 (s, 2H), 4.74 (t, *J* = 9.3 Hz, 2H). ^13^C NMR (75 MHz, CDCl_3_) ∂ ppm: 164.52, 164.18, 163.89, 159.08, 155.60, 155.46, 136.49, 132.14, 131.82, 130.38, 129.97, 128.85, 128.38, 127.73, 126.67, 126.10, 123.08, 122.32, 122.24, 121.36, 115.97, 70.46, 62.20.

**4-{[4-(4,4,6,6,7,7,9,9,10,10,12,12,12-tridecafluoro-2,5,8,11-tetraoxadodecanoyl)phenoxy]-carbonyl}phenyl 3-hydroxybenzoate**

A solution of the benzyl-protected tricyclic phenol (16.60 g, 20 mmol) in CH_2_Cl_2_ (150 mL) and EtOH (150 mL) was first briefly evacuated and purged with argon, then 10% Pd/C catalyst (2.82 g) was added. The argon atmosphere was replaced by hydrogen gas, and the reaction mixture was stirred at room temperature for 3 hours. Hydrogen was pumped out of the system and the flask was purged thoroughly with argon. The mixture was filtered through Celite and solvents were removed under reduced pressure. This gave tetracyclic phenol **8** as white powder with phase sequence: I 163 °C SmA 110 °C X (14.75 g, 99%).

^1^H NMR (300 MHz, Chloroform-d) ∂ ppm: 8.35–8.22 (m, 2H), 8.21–8.10 (m, 2H), 7.81 (ddd, *J* = 7.7, 1.6, 1.0 Hz, 1H), 7.71–7.63 (m, 1H), 7.48–7.30 (m, 5H), 7.15 (ddd, *J* = 8.1, 2.7, 1.0 Hz, 1H), 5.13 (s, 1H), 4.73 (t, *J* = 9.3 Hz, 2H).

^13^C NMR (75 MHz, CDCl_3_) ∂ ppm: 155.98, 155.54, 132.16, 131.83, 130.22, 126.71, 122.94, 122.30, 122.24, 121.36, 116.97, 115.70, 62.23.

**4-{[4-(4,4,6,6,7,7,9,9,10,10,12,12,12-tridecafluoro-2,5,8,11-tetraoxadodecanoyl)phenoxy]carbonyl}-phenyl 3-[4-(benzyloxy)benzoyloxy]benzoate**

To a suspension of compound the tricyclic phenol resulting from the above debenzylation (14.08 g, 18 mmol) and 4-benzyloxy benzoic acid (**4**) (4.21 g, 18 mmol) in CH_2_Cl_2_ (800 mL), DCC was added (4.5 g, 22 mmol), as well as a trace of DMAP. The reaction mixture was allowed to stir at room temperature for 3 days, then filtered, washed with water, 5% CH_3_COOH, water, brine, dried over MgSO_4_, filtered, and concentrated at reduced pressure. The crude product was purified by flash chromatography (silica gel, CH_2_Cl_2_), followed by two crystallizations from CH_3_CN and later from toluene to give a white solid, I 156 °C SmA 150 °C Sm (not assigned) 127 °C Glass (9.92 g, 57%).

^1^H NMR (300 MHz, Chloroform-d) ∂ ppm: 8.36–8.03 (m, 8H), 7.67–7.30 (m, 11H), 7.14–7.03 (m, 2H), 5.18 (s, 2H), 4.73 (t, *J* = 9.2 Hz, 2H). ^13^C NMR (75 MHz, CDCl_3_) ∂ ppm: 164.79, 164.18, 163.87, 163.85, 163.46, 155.44, 151.40, 136.16, 132.59, 132.17, 131.82, 130.60, 129.99, 128.89, 128.47, 127.79, 127.65, 126.78, 126.10, 123.92, 122.29, 122.23, 121.60, 114.98, 70.39, 62.20.

**4-{[4-(4,4,6,6,7,7,9,9,10,10,12,12,12-tridecafluoro-2,5,8,11-tetraoxadodecanoyl)phenoxy]carbonyl}-phenyl 3-(4-hydroxybenzoyloxy)benzoate (8)**

A solution of compound the tetracyclic benzylether-protected phenol from above (9.37 g, 9.67 mmol) in CH_2_Cl_2_ (250 mL) and EtOH (150 mL) was first briefly evacuated and purged with argon, then 10% Pd/C catalyst (3 g) was added. The argon atmosphere was replaced by hydrogen gas, and the reaction mixture was stirred at room temperature for 3 h. Hydrogen was pumped out of the system and the flask was purged thoroughly with argon. The mixture was filtered through Celite and solvents were removed under reduced pressure. This gave the key intermediate tetracyclic phenol **8** as a white crystalline solid mp 170–172 °C.

^1^H NMR (300 MHz, Chloroform-d) ∂ ppm: 8.35–8.10 (m, 8H), 7.67–7.30 (m, 6H), 6.98–6.87 (m, 2H), 5.58 (s, 1H), 4.73 (t, *J* = 9.3 Hz, 2H). ^13^C NMR (75 MHz, CDCl_3_) ∂ ppm: 164.83, 164.21, 163.93, 160.76, 155.43, 151.35, 135.61, 132.88, 132.19, 132.11, 131.83, 130.60, 130.02, 127.85, 126.78, 126.11, 123.92, 122.29, 122.24, 121.60, 115.71, 62.22.

**4-{[4-(4,4,6,6,7,7,9,9,10,10,12,12,12-tridecafluoro-2,5,8,11-tetraoxadodecanoyl)phenoxy]carbonyl}-phenyl 3-[4-(4-cyanobenzoyloxy)benzoyloxy]benzoate (W788)**

Tetracyclic phenol **8** (1.01 g, 1.15 mmol) was dissolved in CH_2_Cl_2_ (150 mL) and cooled to 0 °C. Triethylamine (0.133 g, 1.31 mmol, 0.2 mL) was then added, followed by dropwise addition of 4-cyanobenzoyl chloride (**9**) (0.190 g, 1.15 mmol), followed by addition of a trace of DMAP. The reaction mixture was stirred overnight at room temperature, poured into the saturated NH_4_Cl, and extracted to the CH_2_Cl_2_ (three times). The combined organic layers were washed with water, brine, dried over MgSO_4_, filtered and concentrated at reduced pressure. The crude product was purified by flash chromatography (silica gel, CH_2_Cl_2_/1% EtOAc), then filtered and crystallized from CH_3_CN twice. The result was a white solid (0.750 g. 68 %).

^1^H NMR (300 MHz, Chloroform-d) ∂ ppm: 8.40–8.25 (m, 6H), 8.22–8.06 (m, 4H), 7.92–7.80 (m, 2H), 7.70–7.51 (m, 2H), 7.48–7.30 (m, 6H), 4.73 (t, *J* = 9.2 Hz, 2H). ^13^C NMR (75 MHz, CDCl_3_) ∂ ppm: 164.20, 163.84, 155.43, 155.39, 155.00, 151.14, 132.93, 132.67, 132.25, 132.19, 131.82, 130.89, 130.76, 130.13, 128.09, 127.24, 126.84, 123.78, 122.27, 122.23, 122.11, 117.85, 117.60, 62.20.

HRMS (ESI-TOF) *m*/*z* for C_43_H_22_F_13_NO_13_Li^+^ [M + Li]^+^; calc. 1014.1038, found 1014.1040.

**DSC 5 deg/min:**

Heating: Glass 134.4 °C SmX (ΔH = −1.19 KJ mol^−1^) 169.9 °C I (ΔH = −33.95 KJ mol^−1^).

Cooling: I 167.3 °C Col (ΔH = 8.09 KJ mol^−1^) 157.9 °C SmX (ΔH = 23.86 KJ mol^−1^) 105.5 °C (ΔH = 0.70 KJ mol^−1^).

**4-{[4-(4,4,6,6,7,7,9,9,10,10,12,12,12-tridecafluoro-2,5,8,11-tetraoxadodecanoyl)phenoxy]carbonyl}-phenyl 3-{4-[4-(trifluoromethoxy)benzoyloxy]benzoyloxy}benzoate (W789)**

Tetracyclic phenol **8** (1.34 g, 1.52 mmol) was dissolved in CH_2_Cl_2_ (200 mL), and cooled to 0 °C. Trietheylamine (0.182 g, 1.80 mmol, 0.25 mL) was then added, followed by dropwise addition of 4-trifluoromethoxy benzoyl chloride (**10**) (0.341 g, 1.52 mmol, 0.24 mL), followed by addition of a trace of DMAP. The reaction mixture was allowed to stir overnight at room temperature, poured to the saturated NH_4_Cl, and extracted to CH_2_Cl_2_ (three times). The combined organic layers were washed with water, brine, dried over MgSO_4_, filtered, and then concentrated at reduced pressure. The crude product was purified by flash chromatography (silica gel, CH_2_Cl_2_/1% EtOAc), then filtered and crystallized from CH_3_CN to give **W789** as a white solid (1.25 g, 82%).

^1^H NMR (300 MHz, Chloroform-d) ∂ ppm: 8.39–8.24 (m, 6H), 8.20–8.07 (m. 4H) 7.67–7.57 (m, 2H), 7.45–7.33 (m, 8H), 4.73 (t, *J* = 9.3 Hz, 2H). ^13^C NMR (75 MHz, CDCl_3_) ∂ ppm: 164.30, 164.18, 163.84, 163.79, 163.54, 155.44, 155.40, 155.30, 151.17, 132.52, 132.19, 131.83, 130.74, 130.12, 128.07, 127.71, 127.41, 126.94, 126.83, 126.11, 123.81, 122.28, 122.24, 120.66, 120.64, 62.21.

HRMS (ESI-TOF) *m*/*z* for C_43_H_22_F_16_O_14_Li^+^ [M + Li]^+^; calc. 1073.0909, found 1073.0914.

**DSC 5 deg/min:**

Heating: Glass 123.3 °C SmAP_F_ (ΔH = −29.73 KJ mol^−1^) 196.2 °C I (ΔH = −14.32 KJ mol^−1^).

Cooling: I 194.6 °C SmAP_F_ (ΔH = 15.89 KJ mol^−1^) 98.8 °C (ΔH = 20.29 KJ mol^−1^)^.^
